# Development of a diagnostic protocol for dizziness in elderly patients in general practice: a Delphi procedure

**DOI:** 10.1186/1471-2296-10-12

**Published:** 2009-02-07

**Authors:** Otto R Maarsingh, Jacquelien Dros, Henk C van Weert, François G Schellevis, Patrick J Bindels, Henriette E van der Horst

**Affiliations:** 1 Department of General Practice and Institute for Research in Extramural Medicine, VU University Medical Center, Amsterdam, The Netherlands; 2 Department of General Practice, Academic Medical Center, University of Amsterdam, Amsterdam, The Netherlands; 3 NIVEL, the Netherlands Institute for Health Services Research, Utrecht, The Netherlands; 4 Department of General Practice, Erasmus University Medical Center, Erasmus University Rotterdam, Rotterdam, The Netherlands

## Abstract

**Background:**

Dizziness in general practice is very common, especially in elderly patients. The empirical evidence for diagnostic tests in the evaluation of dizziness is scarce. Aim of our study was to determine which set of diagnostic tests should be part of a diagnostic protocol for evaluating dizziness in elderly patients in general practice.

**Methods:**

We conducted a Delphi procedure with a panel of 16 national and international experts of all relevant medical specialities in the field of dizziness. A selection of 36 diagnostic tests, based on a systematic review and practice guidelines, was presented to the panel. Each test was described extensively, and data on test characteristics and methodological quality (assessed with the Quality Assessment of Diagnostic Accuracy Studies, QUADAS) were presented. The threshold for in- or exclusion of a diagnostic test was set at an agreement of 70%.

**Results:**

During three rounds 21 diagnostic tests were selected, concerning patient history (4 items), physical examination (11 items), and additional tests (6 items). Five tests were excluded, although they are recommended by existing practice guidelines on dizziness. Two tests were included, although several practice guidelines question their diagnostic value. Two more tests were included that have never been recommended by practice guidelines on dizziness.

**Conclusion:**

In this study we successfully combined empirical evidence with expert opinion for the development of a set of diagnostic tests for evaluating dizziness in elderly patients. This comprehensive set of tests will be evaluated in a cross-sectional diagnostic study.

## Background

Dizziness is very common, especially in elderly patients. [1-3] In 2002 almost 10% of patients aged 65 years or older consulted their general practitioner because of dizziness [Maarsingh/Dros et al., Dizziness in elderly patients in general practice: prevalence, incidence and clinical characteristics, submitted]. For clinicians dizziness often represents a diagnostic problem, because it is a subjective sensation that can be caused by a wide range of benign and/or serious conditions.[4,5] In 20–40% of the dizzy patients the underlying cause remains unknown. [6-8]

Although practice guidelines recommend the use of several diagnostic tests in the evaluation of dizziness, these recommendations are mainly expert-based. Many authors have reported on tests used for diagnosing dizziness, but few studies investigated the diagnostic accuracy of these tests. Often the methodological quality of these studies was poor. In addition, all diagnostic accuracy studies were carried out in secondary or tertiary care settings. Therefore these results cannot be straightforward extrapolated to a primary care setting. Furthermore, none of these studies included an elderly population, although the prevalence of dizziness as well as the risk of more serious pathology increases with age [Dros/Maarsingh et al., Dizziness in primary care: a systematic review of diagnostic tests, submitted].

The aim of our study was to determine which set of diagnostic tests should be part of a diagnostic protocol for evaluating dizziness in elderly patients in general practice. This set of tests will be evaluated in a cross-sectional diagnostic study. Because the empirical evidence is scarce and guidelines are contradictory, we have chosen to conduct a Delphi procedure. During this procedure we combined empirical evidence with expert opinion, in order to create a solid base for a future guideline on dizziness.[9]

## Methods

### Sources of evidence (Flowchart: Figure 1)

**Figure 1:**
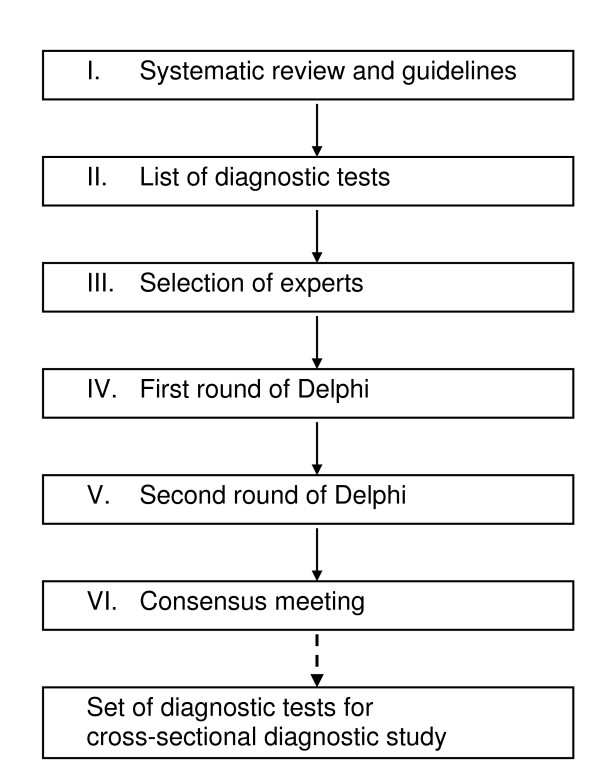
**Flowchart Delphi procedure**.

To identify potentially relevant diagnostic tests for dizziness in general practice we performed a sensitive search in PubMed, EMBASE, PsychINFO, CINAHL and Gerolit, from database inception to February 2005 [Dros/Maarsingh et al., Dizziness in primary care: a systematic review of diagnostic tests, submitted]. Two reviewers (OM and JD) independently selected potentially relevant studies on title and abstract (selection criteria: Appendix 1). From the initial 17,950 hits, 228 studies on diagnostic tests for dizziness possibly feasible in primary care were retrieved for full-text reading. One hundred and eighteen studies were excluded after full-text reading. The methodological quality of the remaining 110 studies was assessed with the Quality Assessment of Diagnostic Accuracy Studies (QUADAS) by two couples of reviewers (OM/HvdH and JD/HvW.[10] We deemed item three (the reference standard is likely to classify the target condition correctly) a crucial feature of the QUADAS-tool. Without an appropriate reference standard misclassification will occur, and therefore study results can be misleading. In addition we searched for practice guidelines on dizziness, syncope, or vertigo (Appendix 2).

A diagnostic test was added to the initial list, to be presented to the panel, if the identified supportive study met at least item three of the QUADAS-tool, or if the test was recommended by a practice guideline on dizziness, syncope, or vertigo. Diagnostic tests that are not feasible in general practice, and/or for which patients must be referred to a specialist (like electronystagmography, or MRI) were removed from the initial list. Based on the identified diagnostic studies and practice guidelines we constituted an initial list of 36 diagnostic tests: 4 elements of patient history, 21 elements of physical examination, and 11 additional tests.

### Study participants

A group of 24 national and international experts on dizziness (representing general practice, geriatric medicine, internal medicine, ENT, nursing home medicine, neurology, cardiology and rehabilitation medicine) were invited to participate in the Delphi procedure. Selection of experts was based on publications (i.e. clinically relevant international publications on dizziness, syncope, or vertigo), or participation in the development of a guideline on dizziness, syncope, or vertigo.

### The Delphi method

The Delphi method, developed by the Rand Corporation in the 1950s, is a method for eliciting consensus opinions from experts.[11] Characteristics of the Delphi method are anonymity (achieved by use of a questionnaire, to avoid dominance of members of the expert panel), iteration (process occurs in "rounds", allowing individuals to change their opinion), controlled feedback (showing the distribution of the group's response), and statistical group response (expressing judgment using summary measures of the full group response).[12] The number of rounds used in the Delphi-process varies, although 2–3 rounds mostly are sufficient. [13-15]

We conducted our Delphi procedure via e-mail. In the first round each participant received the list of 36 potential diagnostic tests, with background information on every test, and an overview of the corresponding empirical evidence (as assessed with the QUADAS-tool). The participants also received background information on the search for evidence, the Delphi procedure, the abstracts of relevant diagnostic studies, and a summary of nine practice guidelines concerning 'dizziness', 'syncope' or 'vertigo'. [16-24] The pdf-file with background information, as it was sent to the participants, can be retrieved by e-mail from the first author. A score form was supplied on which each participant could indicate if a test should be incorporated in a diagnostic protocol for dizzy elderly patients in primary care (yes/no). When participants thought they had insufficient expertise on a particular test (not used in their speciality), they could refrain from answering this question. Participants had to motivate why a test should be excluded from the diagnostic protocol. On the score form we supplied six pre-defined categories for motivation of exclusion. These categories were derived from a framework often used to evaluate diagnostic technologies by categorizing studies into six hierarchical levels[25]: 1. technical feasibility, 2. diagnostic accuracy, 3. diagnostic thinking impact, 4. therapeutic choice impact, 5. patient outcome impact, and 6. societal impact.

If a participant deemed that another diagnostic test should be added, he or she could note this on the score form with a motivation and a brief description of the test.

Tests on which at least 70% of the participants agreed with each other were either included in the protocol or deleted from the list. Tests on which no agreement had been reached were presented in the second round. In this round participants received information on the percentage agreement per test in the first round, motivations for rejection, a summary of comments of the participants, and their own score compared with the group score. The participants had to indicate for each remaining test if it should be incorporated in the diagnostic protocol with a short motivation. This round allowed participants to change their score in view of the group's response.

We planned to carry out additional rounds if necessary. We scheduled four weeks for each Delphi round, two weeks for the participants to complete the score form, and two weeks to interpret the results and to incorporate them into the subsequent round's score form.

## Results

### Expert panel

E-mail invitations explaining the study were sent to 24 experts, of which 16 agreed to participate. Reasons for non-participation were lack of time (n = 5), lack of expertise (n = 1), or unknown (n = 2). Information on the participants is presented in Table 1. All participants completed the full procedure.

**Table 1:** Characteristics of Delphi Respondents (N = 16)

Professional role	5 General Practitioners
	3 Geriatricians
	2 Cardiologists
	2 ENT Specialists
	1 Specialist for Internal Diseases
	1 Neurologist
	1 Nursing Home Doctor
	1 Rehabilitation Specialist
Years of experience in current position*	17.7 (minimum 3 years, maximum 30 years)
Location of participant	The Netherlands = 12
	Finland = 1
	Sweden = 1
	United Kingdom = 1
	United States = 1
Mean number of international publications on dizziness, syncope, or vertigo*	6.5 (minimum 0 publications, maximum 52 publications)

*: At January first, 2006

### Delphi rounds

The results of the Delphi procedure are presented in Table 2.[6,16-24,26-69] The first round resulted in the inclusion of 16 tests and the exclusion of four tests. Most often, the motivation for exclusion of a test was (lack of) technical feasibility (level one), (lack of) diagnostic accuracy (level two), or (lack of) diagnostic thinking impact (level three). In 83 (14.4%) of in total 576 judgements, a participant stated that he or she had insufficient expertise to judge a particular test. One diagnostic test ('alternative' orthostatic test), suggested by one of the experts, was added to the procedure after this first round.

**Table 2:** Results of the Delphi Procedure

**Diagnostic test** **(N = 36+1)**	First round					Second round				Consensus round†
	Inclusion	Exclusion	No expertise	Most important motivation for exclusion*	Result†	Inclusion	Exclusion	No expertise	Result†	Result†

**I. Patient History**[6,16-19,21-24]										
										
1. Present dizzy symptoms	16	0	0	-	**I**					
2. Medication	16	0	0	-	**I**					
3. Alcohol intake	15	1	0	2,5	**I**					
4. Medical history	16	0	0	-	**I**					
										
**II. Physical Examination**										
										
*Cardiovascular System*										
5. Pulse Measurement [16-19,21-23]	13	3	0	3	**I**					
6. Blood pressure [17-19,21-24]	16	0	0	-	**I**					
7. Orthostatic hypotension test[17-19,21,23,24,27,28]	10	6	0	1,3	2	10	6	0	C	**I**
8. Orthostatic test[58]‡	7	8	1	1	2	3	13	0	**E**	
9. Auscultation of the heart[16-19,21-24]	14	2	0	2,3	**I**					
10. Auscultation of the carotids[18,24,49,51]	9	7	0	2	2	10	6	0	C	**E**
Extra test, added after round 1:										
Alternative orthostatic test§						2	12	2	**E**	
*Locomotor System*										
11. Orthopaedic screening of lower extremities[21,22,24]	9	3	4	3	**I**					
12. Toe and heel gait[22]	6	7	3	2,3	2	5	8	3	C	**E**
13. One-leg stance test[21,37]	2	9	5	3	**E**					
14. Tandem gait[21,22,24,37]	10	3	3	2	**I**					
15. Performance-oriented mobility assessment[41,63]‡	1	10	5	1	**E**					
16. Berg Balance Scale [31-33,35,36]‡	5	5	7	3	2	3	8	5	**E**	
17. The timed 'up and go'-test [21,22,55,59]	5	5	6	2	2	5	6	5	C	**E**
*Neurological System*										
18. Tendon reflexes[22,24]	11	2	3	1,2,3	**I**					
19. Semmes-Weinstein Monofilament Test[71,72]‡	7	5	4	3	2	7	5	4	C	**I**
*Vestibular System*										
20. Otoscopy[17,21,22,24]	10	3	3	2,3	**I**					
21. Dix-Hallpike Maneuver [17,20,24,30,34]	10	3	3	1,2	**I**					
22. Side-lying[42]‡	5	8	3	2	2	2	11	3	**E**	
23. Head-shaking Nystagmus [20,39,45,46,48,50,62,64,65]	4	9	3	1,2	2	0	12	4	**E**	
24. Vibration-induced nystagmus[20,44,47,57]	2	11	3	1	**E**					
*Remaining Tests*										
25. Visual acuity[20,22]	11	2	3	1,3	**I**					
										
**III. Additional Tests**										
										
*Cardiovascular System*										
26. Electrocardiogram [16,18,19,21,23,24]	13	2	1	1,2,3,4	**I**					
27. Carotid sinus massage [19,23,40,43,49,51,52,56,66]	3	11	2	1	**E**					
28. ECG-monitoring [18,19,23,29,38,53,54,60,61,67]	10	5	1	1	2	13	2	1	**I**	
*Laboratory Tests*[24,69]										
29. Erythrocyte sedimentation rate[24]	7	7	2	3	2	6	9	1	C	**E**
30. Haemoglobin[24]	13	1	2	3	**I**					
31. Non-fasting blood glucose‡	12	2	2	3	**I**					
32. Serum potassium level‡	9	5	2	3	2	11	4	1	I	**E**||
33. Serum sodium level‡	8	6	2	3	2	10	5	1	C	**E**
34. Thyroid function‡	6	7	3	3	2	5	9	2	C	**E**
*Psychiatric Testing*										
35. Patient Health Questionnaire[68]‡	8	4	4	1,2	2	9	4	3	C	**I**
*Vestibular System*										
36. Audiometry[17,21,22,24,26]	9	4	3	1	2	10	3	3	**I**	

*:  1: Technical feasibility; 2: Diagnostic accuracy; 3: Diagnostic thinking impact; 4: Therapeutic  choice impact; 5: Patient outcome impact; 6: Societal impact  †: I: Inclusion; E: Exclusion; 2: Second round; C: Consensus round; the threshold for respectively in-  or exclusion was set at an agreement of ≥70%  ‡:  Not recommended by any practice guideline on dizziness, syncope, or vertigo  §: Blood pressure measurement after 5 min of lying supine, followed by measurement after standing  for 5 min or when orthostatic symptoms do occur; decrease in systolic blood pressure ≥ 20 mmHg  or a decrease of systolic blood pressure to < 90 mmHg is defined as orthostatic hypotension  ║: Eventually removed, because of the lack of evidence, and the high chance of false-positives, in  combination with the impact for the patient (intra-venous puncture).

The level of agreement in the first round for recommended tests compared to non-recommended tests was 78.5% vs. 71.1%.

In the second round, 17 tests (16 tests remaining from the first round, and the added alternative orthostatic test) were assessed. This resulted in three included tests, five excluded tests, and nine tests on which no agreement could be reached. In total, 19 tests were included after 2 rounds.

Participants changed their opinion on average almost three times out of 17 (17.3%), varying from zero to six times.

### Consensus meeting

Taking into consideration the comments of the participants, and the limited change in group scores of the nine tests on which no agreement had been reached, we deemed an additional voting round not fruitful. The remaining nine tests were therefore summarized (including the panel scores, comments of the participants, and additional scientific evidence) and discussed in a consensus meeting of the research group, after which a draft diagnostic protocol was constructed. Three of the nine tests were added to the draft protocol: the Orthostatic hypotension test (OHT), the Semmes-Weinstein Monofilament Test (SWMT), and the Patient Health Questionnaire (PHQ). Motives for adding the OHT (63% agreement after the second Delphi round) were the frequent application of this test in daily practice (investigating the diagnostic value therefore is useful), the high prevalence of orthostatic hypotension in elderly people, and the strong request of several panel members to include this test. Motives for adding the SWMT (58% agreement) were the lack of somatosensory tests on the list, the user-friendliness, and the fact that GPs are already familiar with this test (as part of diabetes care). The PHQ (69% agreement) was added to the draft protocol, because various studies suggest that psychiatric disorders may play a causative or contributory role in dizziness.[6,69] Therefore a psychiatric evaluation should not be missed in the protocol, as several panel members stated on their forms.

The PHQ and the SWMT were the only included tests that have not been recommended by any existing guideline on dizziness, syncope, or vertigo.

### Removal of tests

Although the diagnostic test serum Potassium level did reach the threshold for inclusion (73% agreement), we eventually removed this test from the draft diagnostic protocol. Motives for removal were the lack of evidence,[69] and the high chance of false-positives,[70] in combination with the impact for the patient (intra-venous puncture). An additional search in Pubmed (("Hyperkalemia" [MeSH] OR "Hypokalemia" [MeSH]) AND (dizz* [tw] OR vertig* [tw])) did not yield relevant publications.

### Draft protocol

The resulting draft protocol contained 21 diagnostic tests. Sixteen tests were included in the first round, three tests were included in the second round, three tests were added during the consensus meeting, and one test was eventually removed. This draft protocol, supplied with the reasoning as mentioned above, was sent to all participants of the Delphi-procedure. They were asked to respond within two weeks if they objected to the added three tests (OHT, SWMT, and PHQ), or if they objected to the removed test (serum Potassium). None of the participants had any objections.

### Final diagnostic protocol

The final diagnostic protocol contained 21 tests, concerning patient history (4 items), physical examination (11 items), and additional tests (6 items), and is shown in Table 3.

**Table 3:** Final diagnostic protocol for evaluating dizziness in elderly patients in general practice

**I. Patient History**
Present dizzy symptoms
Medication
Alcohol intake
Medical history

**II. Physical Examination**
*Cardiovascular System*
Pulse measurement
Blood pressure
Orthostatic hypotension test
Auscultation of the heart
*Locomotor System*
Orthopaedic screening of lower extremities
Tandem gait
*Neurological System*
Tendon reflexes
Semmes-Weinstein Monofilament Test
*Vestibular System*
Otoscopy
Dix-Hallpike maneuver
*Remaining Tests*
Visual acuity

**III. Additional Tests**
*Cardiovascular System*
Electrocardiogram
ECG-monitoring
*Laboratory Tests*
Haemoglobin
Non-fasting blood glucose
*Psychiatric Testing*
Patient Health Questionnaire
*Vestibular System*
Audiometry

## Discussion

In this study we combined empirical evidence with expert opinion for the development of a set of diagnostic tests for evaluating dizziness in elderly patients in general practice.

Five tests were excluded during the procedure, although they are recommended by several practice guidelines: auscultation of the carotids,[18,24] toe and heel gait,[22] one-leg stance test,[21] the timed 'up and go'-test,[21,22] and carotid sinus massage.[19,23] For these five tests, the experts questioned the diagnostic accuracy and the added diagnostic value. For carotid sinus massage the experts also questioned the technical feasibility. By contrast, the diagnostic tests serum haemoglobin level, and capillary non-fasting blood glucose level were included during the procedure, although several guidelines question their diagnostic value.[16,19,21-23,69] Two included tests (SWMT and PHQ) have not been recommended by any practice guideline on dizziness, syncope, or vertigo. Until now, the SWMT has not been tested in a dizzy population. However, it is frequently used for detecting peripheral neuropathy in diabetic patients.[71,72] Because peripheral neuropathy can contribute to complaints of dizziness, especially in elderly patients,[73] the SWMT was part of the initial list of 36 diagnostic tests. The PHQ has been tested only once in a dizzy population.[68] However, the assessment of the methodological quality of this study was relatively high (level 2 QUADAS) [Dros/Maarsingh et al., Dizziness in primary care: a systematic review of diagnostic tests, submitted]. Furthermore, several practice guidelines recommend psychiatric screening during the evaluation of dizziness.[19,21-23,69]

This is the first study to describe the use of a Delphi procedure for the development of a diagnostic protocol for dizziness. The Delphi method has advantages compared to other consensus methods. It is swift, inexpensive, and allows combining the knowledge and abilities of an expert group anonymously.[74,75] Informal methods of reaching consensus are recognised to be prone to domination by powerful individuals, the biasing effects of personality traits, seniority, and the fact that only one person can speak at a time.[74,76]

A strength of this study is the preparation for the actual Delphi procedure. According to the Appraisal of Guidelines for Research and Evaluation (AGREE) instrument we provided the experts with details of the search for evidence, including search terms used, and sources consulted (item 8 of the AGREE instrument), we provided the experts with criteria for including/excluding evidence (item 9), and we clearly described the Delphi technique itself (item 10).[9] Furthermore, by means of an extensive literature search for original diagnostic studies and existing guidelines, followed by the assessment of the methodological quality by QUADAS, we were able to provide the members of the expert panel with a maximum of empirical background information. Another strength is the varied composition of the expert panel (containing eight different medical disciplines).

Our study also has limitations. Firstly, the expert panel has an overrepresentation of Dutch participants (75%). This can affect the selection process, because it's imaginable that participants sometimes respond from the present national point of view. For example, the guideline 'Dizziness' of the Dutch College of General Practitioners advises against laboratory testing,[22] while the guideline 'Vertigo' from Evidence-Based Medicine Guidelines (United Kingdom) advises to examine the Erythrocyte Sedimentation Rate and the blood count.[24] Furthermore, it could be argued that a set of sixteen international experts originating from five different countries is an inappropriate sample to represent experts worldwide. However, for its principle aim, namely to select a set of diagnostic tests for further research, we consider the composition of the expert panel as sufficient. Besides, and probably needless to say, panel members were above all invited because of their estimated competence (based on previous work in the area of dizziness). Another possible limitation is the absence of a consensus meeting with all the members of the expert panel. This might have helped during the construction of the draft diagnostic protocol. However, such a meeting was not possible for practical reasons (large travel distances for the international participants). For future research it could be considered to arrange an internet consensus meeting with all the participants. Furthermore, we emphasize that the initial list of 36 diagnostic tests obviously doesn't cover all available tests for dizziness in general practice. However, members of the expert panel had the opportunity to add a potential missing test during the Delphi-procedure (which only one panel member actually did). Finally, it could be argued that we violated the Delphi procedure by removing the diagnostic test serum Potassium level from the draft protocol, in spite of reaching the threshold. However, we strongly believed that the inclusion of a single invasive diagnostic test with a total lack of evidence, and a high chance of false-positivity was unjustified from a patient point of view.[69,70] This was confirmed by the fact that none of the experts objected to the exclusion of this test during the consensus round.

## Conclusion

In this study we successfully combined empirical evidence with expert opinion for the development of a set of diagnostic tests for evaluating dizziness in elderly patients in general practice. This comprehensive set of tests will be evaluated in a cross-sectional diagnostic study. This should result in a diagnostic strategy that can be incorporated in existing guidelines.

## Abbreviations

QUADAS: Quality Assessment of Diagnostic Accuracy Studies; OHT: Orthostatic hypotension test; SWMT: Semmes-Weinstein Monofilament Test; PHQ: Patient Health Questionnaire.

## Competing interests

The authors declare that they have no competing interests.

## Authors' contributions

HCvW and HEvdH designed this study and obtained the funding. ORM and JD searched for diagnostic tests and guidelines. ORM, JD, HCvW, and HEvdH assessed the methodological quality of identified diagnostic studies. ORM and JD collected the data. All authors participated in the analysis and interpretation of the data. ORM wrote the original draft. All authors revised the draft critically with regard to important intellectual content, and approved the final version of the paper.

## Appendix

Appendix 1: Selection criteria for identifying potentially relevant diagnostic studies on dizziness

a. The title of the abstract includes the word 'dizziness' or 'disequilibrium' or '(pre)syncope' or 'vertigo' or a word with the same meaning or a disease which can cause dizziness.

b. The abstract describes at least one diagnostic test, procedure or strategy.

c. The study population, or at least part of it, has to be 'dizzy'.

d. The study has to be written in English, French, German or Dutch.

e. The study has to be an original study on a diagnostic test, procedure or strategy.

f. The diagnostic test has to be feasible in primary care.

Appendix 2: Search strategy for practice guidelines on dizziness, syncope, or vertigo

I. Pubmed: ("Dizziness" [MeSH] OR "Syncope" [MeSH] OR "Vertigo" [MeSH]) AND ("guideline" [Publication Type] OR "practice guideline" [Publication Type])

II. Dutch internet sites*

- Care4Cure 

- Dutch Association of Insurance Medicine 

- Dutch Association of Nursing Home Physicians 

- Dutch College of General Practitioners 

- Dutch Institute for Healthcare Improvement (CBO) 

- Huisarts en Wetenschap 

- Nederlands Tijdschrift voor Geneeskunde 

- Spreekuurassistent 

- The Netherlands Society of Cardiology (NVVC) 

- The Netherlands Society of Neurology (NVN) 

- The Netherlands Society of Occupational Medicin (NVAB) 

- The Netherlands Society for Otorhinolaryngology and Cervico-Facial Surgery 

III. International internet sites*

- Bandolier 

- Clinical Evidence 

- Clinical Knowledge Summaries 

- The Cochrane Library 

- DARE 

- Das Ärztliche Zentrum für Qualität in der Medizin 

- Evidence-Based Medicine Guidelines 

- Guidelines Finder 

- The Guidelines International Network 

- National electronic Library for Health (NHS), 

- National Guideline Clearinghouse 

- National Institute for Health and Clinical Excellence 

- New Zealand Guidelines Group 

- Scientific Society of Flemish General Practitioners, WVVH 

- Scottisch Intercollegiate Guidelines Network (SIGN) 

- SUMSearch 

- The Swedish Council on Technology Assessment in Health Care 

- Trip Database 

- UpToDate 

*: If an internet site contained hyperlinks to other sites with possible information about practice guidelines on dizziness, syncope, or vertigo, these sites were also visited.

## Pre-publication history

The pre-publication history for this paper can be accessed here:


